# Production and Structural Characterization of *Lactobacillus helveticus* Derived Biosurfactant

**DOI:** 10.1155/2014/493548

**Published:** 2014-11-19

**Authors:** Deepansh Sharma, Baljeet Singh Saharan, Nikhil Chauhan, Anshul Bansal, Suresh Procha

**Affiliations:** ^1^Department of Microbiology, Kurukshetra University, Kurukshetra 136 119, India; ^2^Dairy Microbiology Division, National Dairy Research Institute, Karnal 132 001, India; ^3^Division of Microbiology, Vector Control Research Center, Puducherry 605006, India; ^4^S. A. Jain College, Ambala City 134 002, India; ^5^Department of Chemistry, Kurukshetra University, Kurukshetra 136 119, India

## Abstract

A probiotic strain of *lactobacilli* was isolated from traditional soft Churpi cheese of Yak milk and found positive for biosurfactant production. *Lactobacilli* reduced the surface tension of phosphate buffer saline (PBS) from 72.0 to 39.5 mNm^−1^ pH 7.2 and its critical micelle concentration (CMC) was found to be 2.5 mg mL^−1^. Low cost production of *Lactobacilli* derived biosurfactant was carried out at lab scale fermenter which yields 0.8 mg mL^−1^ biosurfactant. The biosurfactant was found least phytotoxic and cytotoxic as compared to the rhamnolipid and sodium dodecyl sulphate (SDS) at different concentration. Structural attributes of biosurfactant were determined by FTIR, NMR (^1^H and ^13^C), UPLC-MS, and fatty acid analysis by GCMS which confirmed the presence of glycolipid type of biosurfactant closely similar to xylolipids. Biosurfactant is mainly constituted by lipid and sugar fractions. The present study outcomes provide valuable information on structural characterization of the biosurfactant produced by *L. helveticus* MRTL91. These findings are encouraging for the application of *Lactobacilli* derived biosurfactant as nontoxic surface active agents in the emerging field of biomedical applications.

## 1. Introduction

Microbial biosurfactants are structurally diverse group of surface active agents produced by a wide variety of microorganism mainly bacteria, actinomycetes, yeast, and filamentous fungi from different environmental habitats which either adhere to cell surface or produced extracellularly [1–7]. Microbial surfactants are amphiphilic molecules mainly glycolipids, phospholipids, lipopeptides, and polymeric compounds [8–11]. Biosurfactants have diverse chemical structures, compositions, and an extensive variety of applications in dairy, food, beverage, cosmetics, detergent, petroleum, and pharmaceutical industries [12–17].* Bacillus*,* Pseudomonas*, and other genus of soil inhabitant microorganisms are commonly reported for the biosurfactant production but, due to pathogenic nature, their application is limited to only environmental applications [12]. Food, cosmetics, and other therapeutic application of these molecules are still questionable due to nondemonstration of their cytotoxicity and ecotoxicity. A number of studies have reported the potential of* lactobacilli* as biosurfactant producers [1, 2, 16, 18–24]. Information of chemical composition and structure complexity of biosurfactants derived from lactic acid bacteria is inadequate or limited to few reports [21]. Lactic acid bacteria derived biosurfactant have been reported as complex mixture of different composition including carbohydrates, proteins, and glycolipids [13, 19, 21, 23, 25–31]. The main reason that limits its commercial production is the lack of structural and molecular knowledge, so as to use it in pharmaceuticals and food processing sectors. Moreover, to encourage commercial interest, microbial biosurfactants must contest with synthetic surfactants in cost, functionality, toxicity evaluation, and adeptness so that these biomolecules can meet the various applications. The range of substrates available for biosurfactant production is the challenging because it is important to find an appropriate agricultural residue with the right combination of nutrients to support maximal growth and production [1]. Substrates with a high content of carbohydrates meet the requirements for use as inexpensive medium for biosurfactant production. Cheese whey is an example of agroindustrial waste/by-product, with high content of lactose, lipids, and proteins. The present study intends to explore production, structural attributes, thermal stability, and toxicity of biosurfactant produced by the* L. helveticus* MRTL 91 using whey as a conventional substrate.

## 2. Materials and Methods

### 2.1. Microorganism and Its Maintenance

A* lactobacilli* strain isolated from cheese sample (Churpi cheese) was used for biosurfactant production. This strain was found to be biosurfactant producer in a previous study using various appropriate methods (data not shown). The strain was stored at −20°C in MRS broth containing 15% (v/v) glycerol solution. Working agar slants were kept at 4°C for subsequent experiments.

### 2.2. Chemicals and Reagents

All chemicals used in current study were of analytical grade and supplied by Hi-Media Pvt. Ltd., India. Whey was a kind gift from Experimental Dairy Plant, National Dairy Research Institute, Karnal.

### 2.3. Deproteinization of Cheese Whey

Cheese whey was deproteinized after adjusting the pH to 4.5 with 5 N HCl [16]. It was heated at 121°C for 15 min to denature the whey proteins. The precipitates were removed by centrifugation at 4°C and 8000 ×g for 10 min. The supernatant was adjusted to pH 6.7 and sterilized at 121°C for 15 min. Cheese whey permeate was concentrated using reverse osmosis up to approximately 20 g/L of lactose.

### 2.4. Biosurfactant Production in Bioreactor

Biosurfactant production in lab scale bioreactor was carried out in a 3 L fermenter (New Brunswick, USA) with 2 L working volume. The production medium contained deproteinized whey and 10 gL^−1^ yeast extract with controlled pH at 6.2. The fermentation broth was inoculated with 1% (v/v) 18 h old preculture, and the fermentation was carried out for 48 h under batch condition at 37°C. Media was flushed with N_2_ gas to replace dissolved oxygen. Samples for estimation of residual lactose, biomass production, and reduction in surface tension were withdrawn at regular interval during the fermentation.

### 2.5. Bacterial Growth Determination

Bacterial growth was measured by determining the optical density at 600 nm during different time intervals up to 48 h. The biomass concentration (gL^−1^) was determined by weighing cell dry weight. 10 mL volume was filtered (0.22 *μ*m) and left to dry at 105°C for 24 h. All the filters were weighed before filtration and after drying.

### 2.6. Sugar Analysis

Sugar concentration was determined during process by high performance liquid chromatography (Shimadzu, model LC 20AD, Japan) using TSK gel SCX column (Tosoh, Japan) with refractive index detector (model RID-10A). The mobile phase used was 0.01 N H_2_SO_4_ at a flow rate of 0.8 mL/min.

### 2.7. Recovery and Evaluation of Biosurfactant Concentration

Biosurfactant was extracted from biomass with phosphate buffer saline (PBS). The cells were left at room temperature up to 12 h with gentle stirring for biosurfactant release [20]. Surface tension of PBS was regularly measured to confirm release of biosurfactant. Surface tension of supernatant was measured by the du Noüy ring method, using a tensiometer equipped with a 1.9 cm platinum ring at room temperature (Lauda, Germany). The biosurfactant concentrations (gL^−1^) were determined using a calibration curve (surface tension (mNm^−1^) = −8.6465 concentration (g/L) + 76.984, *r* ^2^ = 0.9729). The calibration curve prepared for a commercially available biosurfactant produced by* Pseudomonas aeruginosa* (dirhamnolipid) lowers the surface tension of water to 27 mNm^−1^ from 72 mNm^−1^ [32].

### 2.8. Purification of Biosurfactant

The suspension was dialyzed against demineralized water at 4°C in a dialysis membrane (molecular weight cutoff 10,000 Dalton, Himedia, India) for 36 h and freeze-dried (membrane changed after every 12 h). Dried biosurfactant was stored at 20°C for further experiments. Crude biosurfactant was partially purified in silica gel (60–120 mesh) column eluted with gradient of chloroform and methanol ranging from 20 : 1 to 2 : 1 (v/v). The fractions were pooled after TLC analysis and solvents were evaporated [19].

## 3. Structural Characterization of Biosurfactant

### 3.1. Product Characterization by Thin Layer Chromatography

The composition of the biosurfactant was determined by TLC followed by postchromatographic detection by staining with chromogenic compounds. Briefly, 1 mL aliquot of crude biosurfactant was extracted, concentrated, and resuspended in 5 *μ*L of ethyl acetate and separated on a precoated silica gel plates (Merck, India) using chloroform/methanol/glacial acetic acid (65 : 15 : 2 v/v) as developing solvent system. The sugar moieties were stained with Syldatk reagents (anisaldehyde: sulfuric acid: glacial acetic acid, 0.5 : 1 : 50), whereas the fatty acid moieties were stained with ammonium molybdate/cerium sulfate (0.42%, w/v, ammonium molybdate and 0.2%, w/v, cerium (IV) sulfate in 6.2% sulfuric acid, Rankem, India) and the plates were heated at 120°C for 10 min. The chromatograms of the extracts were compared with the TLC pattern of a standard mixture of rhamnolipids which was prepared from Jeneil JBR 425 (Jeneil Biosurfactants Company, Saukville, USA), containing the dirhamnolipids of* Pseudomonas aeruginosa*. The ionic property of BS was determined by using agar well diffusion method [33].

### 3.2. Liquid Chromatography (UPLC) and Mass Spectroscopy

Separated spots in TLC were dissolved in methanol, and Waters (UPLC) system equipped with quaternary gradient pump, autosampler, and a photo diode detector (PDA, 2996) was used to separate the product accordingly. Separation was performed on C18 column (1.7 *μ*m × 2.1 *μ*m × 100 mm) with column oven temperature held at 40°C. A multistep linear gradient composed of eluent A (water + 0.1% trifluoroacetic acid) and eluent B (acetonitrile + 0.1% trifluoroacetic acid) was applied. The autosampler temperature was maintained at 10°C and 10 *μ*L of sample solution was injected. From 0–13 min a linear gradient was applied from the mixture A : B (70 : 30, v/v) to A : B (0 : 100 v/v). A plateau of 100% eluent B from 13 min to 15 min was set before going back to 70% eluent A from 15 min to 16 min. Flow rate was 0.4 mL/min. The LC system was coupled with a Waters mass spectrometer with an atmospheric pressure electroscopy interface. The ESI source was set in positive and negative ionization mode. Nitrogen gas was used as nebulizer gas and helium gas a collision gas [34].

### 3.3. Fatty Acid Analysis (GCMS)

Sample was reconfirmed on Thermo Scientific TSQ 8000 Gas Chromatograph Mass Spectrometer system equipped with a VF-5MS column. The separation parameters were as follows: the initial column temperature was 100°C for 1 min, then ramped at 30°C to 270°C, and finally held at 270°C for 10 min. The temperatures of the transfer line, ion trap, and quadrupole were 280, 230, and 150°C, respectively. The inlet temperature was 270°C, and a 20 *μ*L sample was injected. The flow rate of the carrier gas (helium) was 1.0 mL min^−1^. After GCMS separation, all the peaks were compared with the standard structural library of fatty acids to determine probable fatty acids composition of the biosurfactant.

### 3.4. FTIR and NMR Structural Elucidation

Fourier transform infrared spectroscopy (FTIR) is used to elucidate the chemical structure of unknown samples by identifying type of functional groups. These infrared absorption bands identify specific molecular components and structures. Infrared spectrum of biosurfactant was recorded on ABB MB-3000 FTIR system by scanning it in the range of 4000–450 cm^−1^ at a resolution of 4 cm^−1^. The purified biosurfactant was dissolved in deuterated chloroform and ^1^H and ^13^C analysis was carried out using Bruker Av II-400 spectrometer. The biosurfactant was dissolved in deuterated chloroform (50 mg mL^−1^) and the spectra were recorded. ^1^H and ^13^C chemical shifts are expressed in ppm relative to the solvent shift as chemical standard.

### 3.5. Thermal Gravimetric (TG) Analysis

Thermal degradation, moisture content, and thermal stability of purified biosurfactant were determined using thermal gravimetric analysis (TGA). Thermal analyses of freeze dried BS were carried out with Mettler Toledo TGA/SDTA system (Greifensee, Switzerland). Briefly, 5–8 mg of lyophilized sample was loaded in a platinum pan and its energy level was scanned in the ranges of 30–480°C and 30–450°C, respectively, under a nitrogen atmosphere, with a temperature gradient of 10°C min^−1^. All the analyses were performed under gradual increase in temperature, plotting the weight percentage and heat flow against temperature respectively.

### 3.6. Phytotoxicity Assay

The phytotoxicity assay of the biosurfactant was determined in a static seed germination and root elongation of the* Brassica nigra* and* Triticum aestivum* slightly [35]. Solutions of biosurfactant were prepared with distilled water at concentration of 0.5 CMC (1.25 mg mL^−1^) and the actual CMC (2.5 mg mL^−1^) and twice the CMC value (5 mg mL^−1^). The seeds were presterilized with sodium hypochlorite. 25 seeds were inoculated in each Petri plate with 10 mL of test solution at 27°C. After five days of incubation in the dark, seed germination, root elongation (>5 mm), and the germination index were recorded as follows:

 relative seed germination (%) = (number of seeds germinated in the extract/number of seeds germinated in the control) × 100;
 relative root length (%) = (mean root length in the extract/mean root length in the control) × 100
 germination index = [(% of seed germination) × (% of root growth)]/100%.

### 3.7. Cytotoxicity Assessment

The cytotoxicity of biosurfactant was checked on mouse fibroblast (ATCC L929) cell line [36]. The cells were cultured in Dulbecco Modified Eagle Medium (DMEM) at 37°C in 5% CO_2_ atmosphere. A standardized quantity of cells (1 × 10^4^) was inoculated in 100 *μ*L of DMEM in 96-well culture plates and incubated for stabilization for 24 h before the treatment. The stock solution of biosurfactant was prepared in DMSO at concentration of 10 *μ*g/1 *μ*L. The final quantities of biosurfactant were added 25 *μ*g, 12.5 *μ*g, and 6.25 *μ*g in the cytotoxicity assay and incubated for 24 h at 37°C in 5% CO_2_ atmosphere. After 24 h, 15 *μ*L dye solution from the CellTiter 96 nonradioactivity cell proliferation assay kit (Promega, USA) was added into the wells and kept for 4 h incubation as per the recommendation. Afterward, the 100 *μ*L stopping solution was added in all the wells and incubated overnight to dissolve formazan product to get uniform readings. The absorbance was recorded at 570 nm in microplate spectrophotometer (molecular devices, SpectraMax, USA). The DMSO used as solvent was taken as negative control in the assay. To estimate the cytotoxicity of biosurfactant, biologically originated rhamnolipid and Sodium dodecyl sulfate (SDS) were used as positive controls.

## 4. Results and Discussion

### 4.1. Production of Biosurfactant

**Figure 1:**
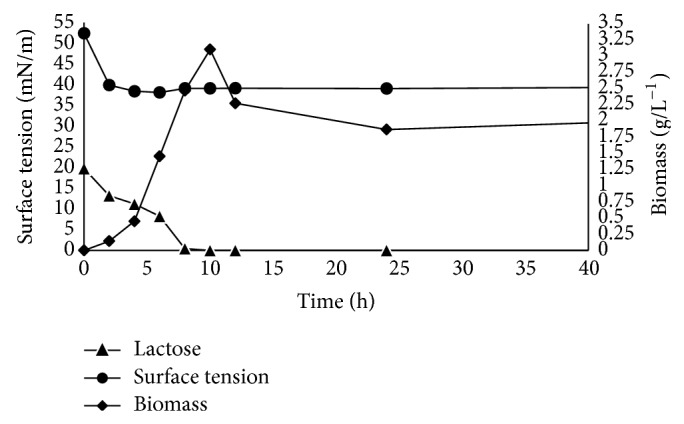
Experimental data of extracellular surface tension variation, biomass, and lactose concentration obtained from fermentation.

Strain MRTL91 was found putative biosurfactant producer. The lowest value of surface tension was achieved after 10 h of fermentation in the stationary phase (39.5 mNm^−1^). The decrease in surface tension was compared with the surface tension of production medium, that is, whey (53.5 mNm^−1^). Samples were withdrawn at regular intervals and experimental data of biomass, lactose consumed, and measurement of surface tension plotted in Figure 1. Lactic acid bacteria (LAB) drastically decrease the pH of the fermentative media by producing lactic acid and other metabolites during the fermentation. Biosurfactant production was found to be growth-associated in shake flasks experiments. The controlled pH at 6.2 positively contributed for higher biomass with maximum utilization of lactose within 10 h after inoculation. The lactose present in the whey was exhausted in first 24 h, further incubation results in cell death. Biomass was measured and found maximum 3.12 g/L^−1^ and the surface tension was reduced down to 39.5 mNm^−1^. Biosurfactant concentration was found to be approximately 0.80 gL^−1^. Increase in initial lactose concentration yields higher biomass and biosurfactant produced by different* lactobacilli* [20]. Biosurfactant produced by* Lactobacillus paracasei* subsp.* paracasei* was also found to be growth-associated. Biosurfactant concentration production was reported to be maximal at stationary growth phase. The similar pattern of biosurfactant production and lactose utilization was also reported [16]. The lowest value of surface tension was achieved in the stationary phase (39.5 mNm^−1^). The results obtained by* L. helveticus* MRTL91 confirmed that the strain is a significant biosurfactant producer. And whey based medium can be used as an alternative substrate for large scale production of biosurfactant.

### 4.2. Structural Characterization of Biosurfactant

**Figure 2:**
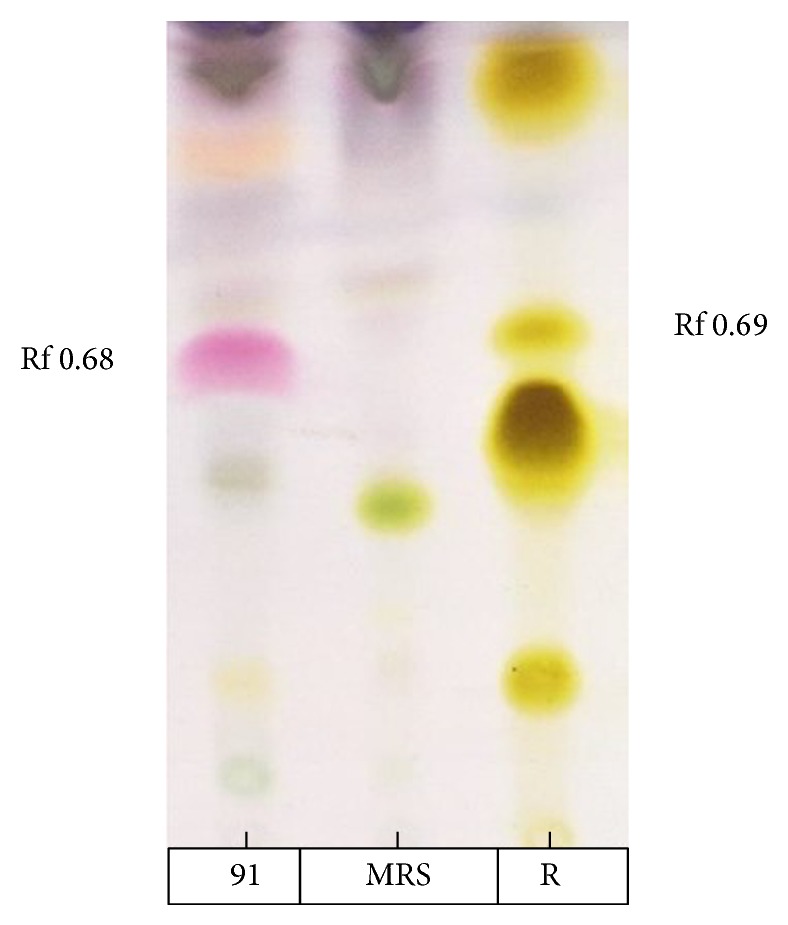
Glycolipid stained with postchromogenic compound (anisaldehyde solution).

**Figure 3:**
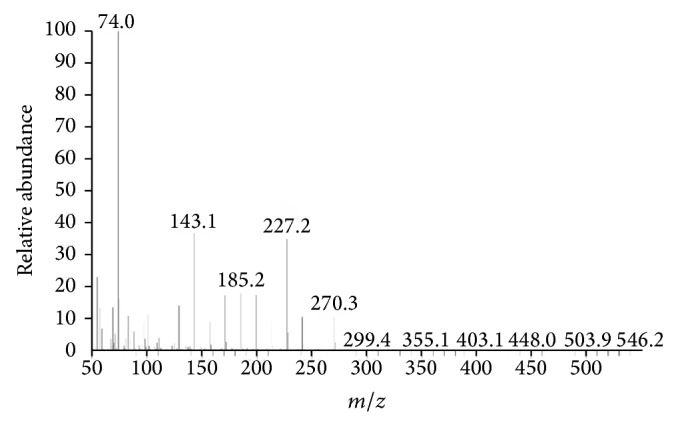
Spectra showing octadecanoic acid as a major fatty acid.

**Figure 4:**
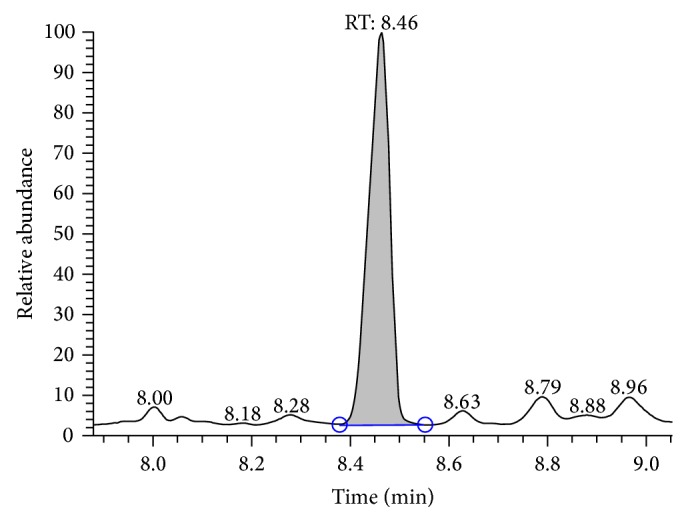
Spectra showing octadecanoic acid separated at retention time of 8.46 min.

**Figure 5:**
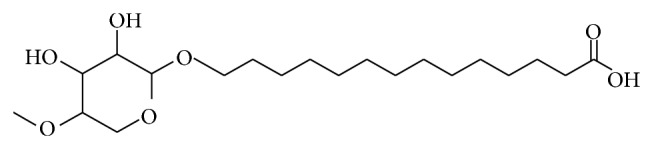
Structure of biosurfactant predicted from IR and NMR (^1^H & ^13^C), LCMS, and GCMS spectrum.

Information obtained from TLC confirmed the presence of glycolipids with polysaccharides and lipid fractions. Biosurfactant was separated with an Rf value of 0.68 as compared to the standard rhamnolipids with an Rf value, that is, 0.69 [36]. Important property of a biosurfactant is its potential to act in the formation of micelles [37, 38]. Surface tension decreases with the increase in biosurfactant concentration and micelles are formed. Critical micelle of biosurfactant produced by strain MRTL91 was found 2.5 mg mL^−1^ which is close to the CMC of synthetic SDS, that is, 1.8–2.9 mg mL^−1^, which reduced surface tension from 72.0 to 37 mNm^−1^ [39]. An effective surfactant can reduce the surface tension of water from 72.0 to 35.0 mNm^−1^ [40]. Biosurfactant obtained from* L. helveticus* MRTL91 showed a significant surface tension reduction as compared to the PBS from 72 to 39.5 mNm^−1^.* Lactobacillus fermentum *RC-14 potentially reduced the surface tension by 72.0 to 39 mNm^−1^ [31].* Streptococcus thermophilus* and* Lactococcus lactis *53 reduce surface tension around 36.0-37.0 mNm^−1^ [24]. Results of present study are in conformity with previous studies of biosurfactants isolated from other LAB strains. Although several reports have been published on biosurfactant produced by LAB, inadequate information is known about their chemical composition. They were characterized as multicomponent mixtures consisting of protein fractions, polysaccharides, and phosphate groups [22, 24, 29, 31, 41]. A glycolipid-like moiety was reported with potent surface active molecule, which reduced the surface tension of PBS water from 72.0 to 39.5 mNm^−1^. The crude biosurfactant was initially characterized by TLC which revealed single spot when being visualized under UV light, which confirmed the presence of glycolipid (Figure 2). The replica plate when stained with iodine vapors produced a dark yellow spot indicating the presence of lipid component. The molecular composition of the crude biosurfactant was evaluated by FTIR, which revealed the presence of polysaccharides and lipid in combination. The most significant bands were located 3456 and 3286 cm^−1^ (for the O–H stretching). The compound showed the C–H stretching vibrations in the transmittance range 2932 cm^−1^ indicating the aliphatic chain. 1720 cm^−1^ (for the C=O ester bond) and 1273 cm^−1^ were found to be ether and C–O stretching vibration in sugars, 1041 cm^−1^ (polysaccharides), 702 cm^−1^, and 648 cm^−1^ (for CH_2_ group) confirming the presence of glycolipid moieties. Biosurfactant produced by* L. helveticus* has been chemically characterized. Results of TLC, FTIR, ^1^H NMR, ^13^C NMR, and GCMS spectra suggest that it consists of several compounds such as octadecanoic acid as main lipid consisting of long aliphatic chain and polysaccharides. Proton and carbon NMR analysis confirmed the presence of –CH_3_ (0.896 ppm), –(CH_2_)_*n*_– (1.286 ppm), –(CH_2_–COO)– (2.324 ppm), –O–CH– (4.386 ppm), and –CH_2_=CH– (7.535 ppm) (Figures 1, 2, and 3). Similar peaks for functional groups were also assigned to the biosurfactant obtained from* Lactococcus lactis*. Proton NMR of* Lactococcus lactis* also showed the similar peaks for spatial arrangement of hydrogen atom [21]. Proton NMR confirmed the presence of carboxyl, alkyl, methyl, alkanes, and keto groups. All spectra showed similarity with the xylolipid reported from other LAB [22]. Purified biosurfactant of* L. helveticus* MRTL 91 was appeared as white powder and found to be anionic in nature. Liquid chromatography and mass spectroscopy also revealed that the biosurfactant is a glycolipid that closely resembles xylolipid previously obtained from LAB. Biosurfactants produced by* Streptococcus mitis* BA and* S. mitis *BMS are composed of extremely low levels of proteins, and the main constituents were glycolipids. Acid precipitated fraction from the* S. mitis* biosurfactant and was characterized as rhamnolipid-like molecules which reduced the surface tension of water to 35 mNm^−1^ at a concentration of 1 mg mL^−1^; on the other hand, crude biosurfactant reduced the surface tension to approximately 48 mNm^−1^ at the same concentration [42]. FTIR, NMR (^1^H and ^13^C), and GCMS confirmed the presence of octadecanoic acid containing glycolipid with a cumulative molecular weight of 391.32* m/z*. GCMS analysis of biosurfactant showed major peaks for octadecanoic acid, a fatty acid at a retention time of 8.46 min (Figures 3 and 4). Gas chromatography-mass spectrum analysis of biosurfactant from* L. helveticus *showed major peaks for octadecanoic acid as a major fatty acid present in biosurfactant.  Figure 5 explained the predicted structure of xylolipid produced by* L. helveticus*. The structure of biosurfactant was also drawn using ChemDraw ultra software. Biosurfactant produced by strain* L. helveticus* is characterized as xylolipid composed of Xylopyranoside with octadecanoic fatty acid chain.

### 4.3. Thermal Gravimetric Analysis (TGA)

**Figure 6:**
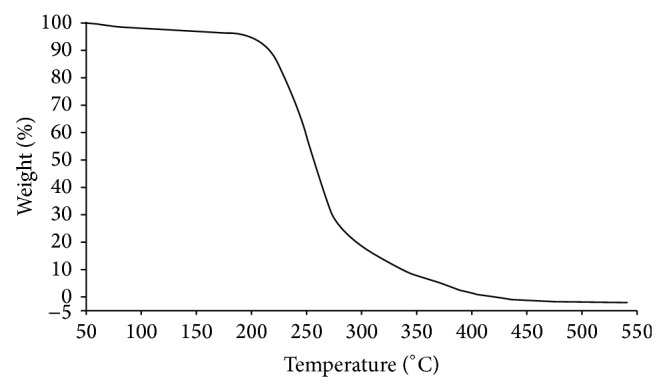
Thermal degradation analysis of BS produced by the* L. helveticus* MRTL91.

Thermal stability of BS is a significant property for its commercial application at extreme temperature. Thermal degradation of BS was carried out by TG analysis (Figure 6). Approximately 1% of weight loss was recorded from increase in temperature from 50 to 220°C possibly due to loss of solvents and moisture molecules. Complete loss of BS was observed after 275°C. It was previously reported that the BS produced from alkalophilic strain of* Klebsiella* spp. showed maximum degradation at 350–400°C [43]. Moisture released during heating of the polymer suggested that the polymer was not truly anhydrous. Similar reports were also reported while working on the rhamnolipid produced by* Pseudomonas aeruginosa *MA01 [44]. The degradation temperature (*T*_*d*_) was 250°C determined from TGA curve. The weight of polymer was drastically lost around and above 290°C and continued gradually to decrease [44]. BS isolated from the strain MRTL 91 shows similar thermal degradation properties close to the rhamnolipids. Regarding the stability at different temperatures (data unpublished), the biosurfactant remained stable after incubation for 120 h to temperatures from 25 to 60°C, with practically no apparent loss of activity. As molecular mass determined by mass spectroscopy confirmed that the BS isolated in the present study has similar molecular mass close to the glycolipid biosurfactant and also exhibited similar thermal degradation properties.

### 4.4. Phytotoxicity and Cytotoxicity of Biosurfactant

Biosurfactant produced by* L. helveticus* was found noncytotoxic and nonphytotoxic. Microbial surface active agents are generally regarded as less toxic and biodegradable biomaterial [3]. But due to huge demand, application of microbial surfactants requires toxicity evaluation before going to be commercialize. In present study, biosurfactant was produced by a GRAS status microorganism and its phytotoxicity and cytotoxicity should be evaluated for its possible application as food ingredients. The germination test has been employed in phytotoxicity assays due to its low implementation cost. Tests including plants are based on seed germination, root growth, root elongation, vigor index, and seedling growth and plants that are profound to toxic matters can be used as bioindicators [45]. The literature reports that some surfactants have an inhibitory effect on plant growth [46].

Various studies have been carried out to find out the toxicity of biosurfactant on seed germination and other vital growth parameters [35, 47–49]. The results obtained in the present study indicate that the solutions tested did not show any inhibitory effect on seed germination/root elongation. The seed germination, root elongation, vigor index, and germination index were used to determine the phytotoxicity of the biosurfactant to the seeds of* Brassica nigra *L. and* Triticum aestivum* L. Different concentration of biosurfactant was prepared at concentration equal to the half of critical micelle concentration (CMC) value, equal to CMC, and twice the CMC. In present study, about 100% seed germination was observed in both types of seeds. But seed germination was declined in the treatment of seeds with SDS (amount equal to CMC) (Tables 1 and 2). Biosurfactant was found less toxic at its CMC concentration as compared to the chemically synthesized SDS. Root elongation, vigor index, and germination index were found better in case of biosurfactant treatment. Root elongation, germination index, and vigor index were found increasing with the increase in concentration of biosurfactant. But, in case of SDS treatment, seed germination, root elongation, germination index, and vigor index were declined as compared to the control treatment of distilled water.

**Figure 7:**
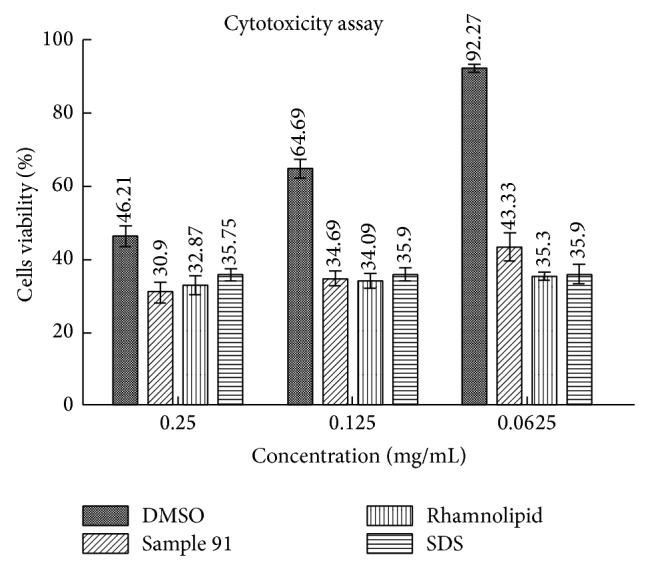
Cytotoxicity evaluation of biosurfactant at different concentration of biosurfactant.

Cytotoxicity of BS was evaluated using mouse fibroblast (ATCC L929) cell line. The mouse fibroblasts cells were selected and generally regarded suitable for cytotoxicity assessment. Mouse fibroblast cells are recommended for in vitro evaluation of medical devices by the International Organization for Standardization (2009). During cytotoxicity determination, different concentrations of cell bound BS and purified rhamnolipids (Janeil, USA) were prepared in DMSO (Figure 7). Whereas SDS at equal concentration was used as negative control, SDS has been admired as a reference irritant because of being fast acting, being nonallergenic, and its toxicity. Significant differences in cell viability of mouse fibroblasts cell were observed at concentrations of 0.25 mg mL^−1^, 0.125 mg mL^−1^, and 0.0625 mg mL^−1^. Cell viability was found maximum about 43.3% at 6.25 mg mL^−1^ in case of biosurfactant produced by strain MRTL91 while positive control rhamnolipid showed 35.3% viability quiet close to SDS, that is, 35.99%. But increase in the concentration of BS also declined the cellular viability. At concentration of 25 mg mL^−1^, cell viability was found 30.9% as compared to rhamnolipid which showed 32.87% of cell viability. While DMSO used as diluent did not show any significant cytotoxicity, the highest biosurfactant concentration studied showed a significant decrease of the total number of viable cells, probably due to a prevalence of a detergent-like effect leading to cell membrane disruption [50]. It is interesting to know that cytotoxicity of biosurfactant produced by strain MRTL 91 showed approximately similar toxicity as compared to the rhamnolipids and SDS.

Various studies on evaluation of cytotoxicity of biosurfactant reported in literature, the lack of cytotoxicity is anticipated when you wish to formulate ecofriendly and safe antiadhesive suspension directly to be used for human health. Typically, the cytotoxicity seems linked to its interactions with the phospholipids of cell membrane and therefore cell lysis. Cochis et al. [36] have reported biosurfactants cytotoxicity on mouse fibroblast cell line with concentrations ranges from 25 to 6.25 *μ*g mL^−1^. Biosurfactant produced by* Sphingobacterium detergens *was studied for its cytotoxicity and antiproliferative effects on different cell lines. When comparing cytotoxicity values (IC_50_) of the two fractions in fibroblast and keratinocyte cell cultures, fraction B was found less cytotoxic, showing lower toxicity than the reference compound SDS, indicating low skin irritability [51]. According to the outcomes of present study, BS produced by* L. helveticus* would be ideal for potential application in cleaning/coating material for several biomedical equipment and cosmetic formulations.

## 5. Conclusion

The identification and structural characterization of new biosurfactant is gaining interest from the commercial point of view. The BS produced by* L. helveticus* MRTL 91 was isolated and structurally characterized as being similar to xylolipid. The FTIR and NMR analysis of biosurfactant revealed the presence of sugar and lipid fractions. Structurally the BS is characterized as a glycolipid with hexadecanoic fatty acid (C16) chain. The minimum surface tension and the CMC were found similar to the previous reports of biosurfactant produced by other* lactobacilli*. Their potential application in products for human consumption such as cosmetics and pharmaceuticals or food additives requires an accurate characterization of possible toxic side effects. Biosurfactant was confirmed as nonphytotoxic and noncytotoxic compound as compared with other microbial and chemically synthesized surface active agents. This is the first compilation of the information on* L. helveticus* derived biosurfactant, structural elucidation, and toxicity assessment. Biosurfactant from LAB, that is, GRAS status organism, is safe for oral consumption and biomedical applications. Structural elucidation opens new horizon for biosurfactants applications in pharmaceuticals/cosmetics and suitable alternative to conventional antimicrobials and antimicrobial resistance.

## Figures and Tables

**Table 1:** Phytotoxicity evaluation of biosurfactant at different concentrations on *Brassica nigra*.

Biosurfactant concentration	*Brassica nigra *			
	Seed germination	Root elongation	Germination index	Vigor index
1.25 mg/mL (1/2 CMC)	100 ± 0.2	105 ± 0.23	105 ± 0.23	1450 ± 74
2.5 mg/mL (CMC)	100 ± 0.1	112 ± 0.22	112 ± 0.21	1475 ± 79
5 mg/mL (2 × CMC)	100 ± 0.15	119 ± 0.15	119 ± 0.19	1525 ± 82
Distilled water	100 ± 0.1	125 ± 0.19	125 ± 0.1	1620 ± 61
SDS (2 mg/mL)	20 ± 0.2	20 ± 0.3	20 ± 0.5	200 ± 58

**Table 2:** Phytotoxicity evaluation of biosurfactant at different concentrations on *Triticum aestivum*.

Biosurfactant concentration	*Triticum aestivum *			
	Seed germination	Root elongation	Germination index	Vigor index
1.25 mg/mL (1/2 CMC)	100 ± 0.2	110 ± 0.2	110 ± 0.34	1600 ± 112
2.5 mg/mL (CMC)	100 ± 0.1	116 ± 0.34	116 ± 0.2	1620 ± 110
5 mg/mL (2 × CMC)	100 ± 0.15	125 ± 0.10	125 ± 0.15	1670 ± 89
Distilled water	100 ± 0.1	126 ± 0.23	126 ± 0.19	1750 ± 76
SDS (2 mg/mL)	20 ± 0.2	25 ± 0.35	25 ± 0.12	250 ± 89
